# Survey data on consumer behaviour in olive oil markets: The role of product knowledge and brand credence

**DOI:** 10.1016/j.dib.2018.04.084

**Published:** 2018-04-30

**Authors:** Melania Salazar-Ordóñez, Macario Rodríguez-Entrena, Elena R. Cabrera, Jörg Henseler

**Affiliations:** a Universidad Loyola Andalucía, Department of Economics, Córdoba, Spain; b Universidad de Cordoba, WEARE - Water, Environmental and Agricultural Resources Economics Research Group, Department of Agricultural Economics, Córdoba, Spain; c Institute of Agricultural Research and Training (IFAPA), Department of Agricultural Economics and Rural Studies, Córdoba, Spain; d University of Twente, Department of Design, Production and Management, Enschede, The Netherlands

## Abstract

This paper presents data conducted to analyse consumer behaviour in agri-food markets, where product differentiation failures occur, with the aim of disentangling the roles played by both consumer information and inferences made from informational stimuli. We thus examined consumer knowledge structures and brand credence related to attitudes towards a particular foodstuff and a product alternative, as well as the actual consumption of the foodstuff. To do so, the selected case study was the olive oil markets in Spain, given that products such as extra virgin olive oil (EVOO) and refined olive oil (ROO), that differ in terms of intrinsic features, become undifferentiated. The data of the observed variables were collected from 700 regular buyers from an online panel at the household level in southern Spain. The data were processed using both Excel for checking, cleaning and descriptive purposes and ADANCO 2.0 (Dijkstra and Henseler, 2015) [1] for performing the model estimations.

**Specifications table**

**Table:** 

Subject area	*Agricultural Economics, Social Science*
More specific subject area	*Consumer behaviour*
Type of data	*Tables, text file*
How data was acquired	*Online survey*
Data format	*Analyzed*
Experimental factors	*Not applicable*
Experimental features	*Not applicable*
Data source location	*Big cities – more than 100,000 inhabitants – in the Andalusia region (Southern Spain)*
Data accessibility	*Data is confidential until the end of the administrative lifespan of the research project*

**Value of the data**

• The data provide a framework about how to build different latent variables using both the composites and common factors paradigms of measurement.
• The data can help to check a theoretical model, which tries to shed light on why product differentiation strategies do not succeed in some agri-food markets.
• The data allow for making a first approximation of those specific factors influencing consumers’ issues in discerning between product features.
• The data provide information about not only factors widely used in the literature of consumer behaviour such as attitude but also some innovative factors related to beliefs regarding a product alternative.
• Until these data, there was no specific information on olive oil markets that analyses, as product alternatives, both extra virgin olive oil (EVOO) and refined olive oil (ROO).

## 1 Data

The primary data were collected using a structured online questionnaire with the aim of assessing a theoretical model about consumer behaviour in agri-food markets with product differentiation failures. The case study was of olive oil markets in southern Spain. Spain is the top olive oil producer worldwide [2], and southern Spain produces 83% of total olive oil in Spain [3]. In addition, its consumption is the second largest worldwide with approximately 11.4 kilos per person per year in the 2013/2014 season [2]. There are two main types of olive oils: “extra virgin olive oil” (EVOO) and “olive oil”, which is composed of refined olive oils and virgin olive oils (ROO). Both products differ significantly in terms of quality, composition and organoleptic properties, EVOO being the highest objective quality category. However, in Spain, ROO is the top-selling type of oil with a 60% market share [4], although the price gap between both has been, on average, around €0.35 kg^−1^ since 2007/2008.

## 2 Experimental design, materials and methods

After the design of the theoretical model, the online questionnaire was structured into five sections in order to collect data about socioeconomic features and 26 observed variables, which were grouped into 8 latent variables. Those latent variables were (see Table 1): Consumption, Attitude towards the main product (EVOO), Attitude towards the product alternative (ROO), Actual knowledge, Self-perceived knowledge, Brand awareness/associations (to the product alternative, ROO), Brand perceived quality (to the product alternative, ROO), and Brand loyalty (to the product alternative, ROO). In this regard, the questionnaire began with general information about the study. After that, respondents pinpointed if they were responsible for food shopping in the household (otherwise, they were discarded). Then, a section was designed to identify the quantities, habits and uses of olive oils and other seed oils, adapting some questions from Saba and di Natale [5]. Once the olive oil category or categories consumed by the respondents were determined, the following sections were customized according to each consumer's consumption pattern. Attitudes towards the most preferred olive oil category were asked about first, and then the opinion about the other category was asked. Again, some questions were adapted from Thorsdottir et al. [6]. In a similar way, the most consumed, known, or preferred brand among the four most well-known leading brands of ROO, whose market share is 27% [7], was inquired about. Brand credence was characterized by means of brand equity components [8] for the abovementioned brands, according to each consumption pattern. On the other hand, questions about product knowledge were proposed in a homogenous style for the whole sample, taking questions from Fotopoulos and Krystallis [9], Torres-Ruíz et al. [10], Brucks [11] and House et al. [12]. The questionnaire finished with a section of sociodemographic data such as gender, schooling, and age. All the observed variables were measured using a 7-point Likert scale, except those belonging to Consumption and Actual knowledge, together with one belonging to Self-perceived knowledge. The observed variables, which define the consumption latent variable, were estimated from self-reported objective actions of consumption in both quantities and habits. For the former, information on the size of the pack and the frequency of purchase of EVOO, ROO, and oil seed was requested; then, the relative amount of EVOO to the total quantity of oils used per capita on a monthly basis for each household was calculated. For the latter, the number of days per week cooking with each type of the abovementioned oils during breakfast, lunch or dinner were used to estimate the relative uses of EVOO to the total habits. The underlying principle of inquiring through different measurement instruments about the consumption patterns was aimed to build a composite reliable latent variable. Regarding the observed variables of Actual knowledge, they were conceptualized as a composed index—composite latent variables—compiling consumers’ objective knowledge measures. Thus, 5 general knowledge questions about the product were made, with the respondents answering “true”, “false”, or “I do not know”. Self-perceived knowledge was also considered a composite. The traditional question about Self-perceived knowledge was measured on a Likert scale (7 points). However, given the composite nature of this latent variable, we also tried to quantify another different aspect of that subjective knowledge. Therefore, we asked a key question, given its complexity for the general public and relevance to the product's quality, in order to get a higher degree of interviewees’ self-perception of their own knowledge: do interviewees know what the olive oil refining process does? The respondents answered either “I know” or “I do not know”. Later, we asked them to explain, in their own words, what the refining process does, by means of an open-ended question. Cramer's V coefficient between respondents’ self-reported knowledge about this issue and their correct or incorrect explanations of the process was 0.26. In addition, approximately one-third of the sample associated the refining process with a practice that improves the intrinsic quality of the olive oil, making it purer, which is totally incorrect. The rest of the latent variables (Attitudes, Brand awareness/associations, Brand perceived quality and Brand loyalty) were considered, following the traditional psychometric approach, as common factors.

**Table 1:** Latent and observed variables.

Latent Variables		Observed Variables	Measurement
Consumption (EVOO)		Cn_1_: Relative consumption of EVOO	% Per cap./monthly
		Cn_2_: Relative uses for cooking with EVOO	% Per household/weekly
Attitude towards the main product (EVOO)		A^product^_3_: The degree to which you need EVOO is…	Likert-scale (1 – the lowest level and 7 – the highest level)
		A^product^_4_: The degree to which you feel EVOO is good for you is….	
		A^product^_5_: The degree to which you would recommend EVOO is….	
		A^product^_6_: The enjoyment you get from the consumption of EVOO is…	
Attitude towards the product alternative (ROO)		A^alternative^_7_: The degree to which you need ROO is…	Likert-scale (1 – the lowest level and 7 – the highest level)
		A^alternative^_8_: The degree to which you feel ROO is good for you is….	
		A^alternative^_9_: The degree to which you would recommend ROO is….	
		A^alternative^_10_: The enjoyment you get from the consumption of ROO is…	
Knowledge	Actual knowledge	Ak_11_: ROO is the superior category of olive oils	True/False/Not sure
		Ak_12_: The taste of EVOO is always strong and bitter	
		Ak_13_: ROO contains refined olive oil plus EVOO	
		Ak_14_: I know at least one olive oil variety (name it)	
		Ak_15_: I know at least one olive oil Protected Designation of Origin (name it)	
	Self-perceived knowledge	Sk_16_: How knowledgeable are you about the features of and matters concerning olive oil?	Likert-scale (1 – the lowest level and 7 – the highest level)
		Sk_17_: Do you know what the olive oil refining process does?	I know/I do not know
Brand equity (to the product alternative, ROO)	Brand awareness/associations	Ba_18_: I can recognize (leading brand) among other competing brands [including my brand]	Likert-scale (1 – the lowest level and 7 – the highest level)
		Ba_19_: When I think of olive oil brands, I have no difficulty in imagining (leading brand) in my mind	
		Ba_20_: I am aware of (leading brand) standing out among other competing brands [including my brand]	
	Brand perceived quality	Bq_21_: The likelihood that (leading brand) quality will never disappoint me [in comparison to alternative brands including my brand] is…	Likert-scale (1 – the lowest level and 7 – the highest level)
		Bq_22_: The likely image of quality of (leading brand) [in comparison to alternative brands including my brand] is…	
		Bq_23_: (Leading brand) has a higher quality in comparison to alternative brands including my brand…	
	Brand loyalty	Bl_24_: The likelihood that (leading brand) is a purchase choice is…	Likert-scale (1 – the lowest level and 7 – the highest level)
		Bl_25_: I would recommend my family and friends buy (leading brand) among other competing brands	
		Bl_26_: Even when another brand is cheaper, I would prefer the (leading brand)	

In the following step, the sample was defined. To do so, the geographical concentration and economic relevance of olive groves in the study region was taken into account. Therefore, the target population included people over 19 years old from large-sized cities—more than 100,000 inhabitants—accounting for 2.4 million people, which comprise 37% of the population [13] in the Andalusia region (southern Spain). Small and medium-sized cities (fewer than 100,000 inhabitants) were discarded since most of them are located in olive oil producing areas, and our focus was on ordinary urban consumers.

Previously, in order to launch the final questionnaire, two experts in olive oil consumption at the national level revised the questionnaire, and two pre-tests were performed to detect potential biases in comprehension. The first one was carried out by means of 30 face-to-face surveys during May 2015; the second one was comprised of 46 respondents from a web-based survey administered during September 2015. The final version of the questionnaire was administered to a sample of 700 regular buyers responsible for household shopping who were over 19 years old. Respondents were drawn randomly from a large on-line internet panel of households maintained by a Spanish-based panel provider from January to September 2016. One of the main drawbacks of using online panels is the fact it is only possible to obtain a sample of the population with access to the internet. Additionally, on-line panels tend to underrepresent some population profiles [14] such as older seniors and lower levels of schooling given the limited use of the internet in those groups. However, according to Baker et al. [15], these panels are particularly meaningful for studying the influence of personal traits such as attitudes, behaviour or intentions; additionally, they minimize missing values. Nonetheless, with the purpose of avoiding under-representation, the sampling was controlled by age and schooling level according to Andalusian regional data [13]. Table 2 shows a descriptive analysis of the sample and population. The statistical power associated to the final sample size (700 respondents) was 87% (*t* test - two tails), considering two predictors, a significance level of 1% and an effect size of 0.02−*f*^2^.

**Table 2:** Descriptive analysis of sample and population.

	Characteristics	Sample (%)	Population (%)^a^	*χ*^2^ test^b^
Gender	Female	52.7	51	*χ*^2^=0.11 (*p*=0.73)
Age	20–39 years	34	36.4	*χ*^2^=1.80 (*p*=0.40)
	40–54 years	35	28.6	
	55+ years	31	35	
Schooling level	University studies	27.4	25.7	*χ*^2^=0.14 (*p*=0.70)
Household members	1–2 people	44		
	3–5 people	53.7		
	5+ people	2.3		
Household income (€ per month)	<1000	19.2		
	1000–2000 €	47.4		
	2001–3000 €	21.9		
	3000+ €	11.5		
Some time living in rural areas	Yes	24.7		

a Data from the Census [16].

b The *χ*^2^ values do not exceed the critical values – $\chi_{1,0.05}^{2}$=3.841; $\chi_{2,0.05}^{2}$=5.991.

Regarding the consumer profiles from the survey, 36% of respondents consumed two or three types of oils, buying bottles of 5 l of EVOO (60% of the sample). Bearing in mind the brand, classification was made taking into account leading brands (product brands from the top four selling companies), cooperative-product brands, and store brands. Therefore, for the purchases of EVOO, consumers preferred cooperative-producer brands in 61% of the cases, and only 11% bought leading brands, while for ROO, they tended to buy leading brands (30%).

As we mentioned above, Consumption, Actual Knowledge and Self-perceived knowledge were thought of as composite latent variables [17]. As a consequence, multicollinearity among indicators should be previously analysed given that high levels of multicollinearity may lead to indicators' unexpected signs or unsuitable confidence intervals [17] in the estimations. Table 3 shows the figures of the variance inflation factors (VIF) for the observed variables of each composite latent variable where there is no such issue, i.e., VIFs are lower than the threshold of 3.3 [18].

**Table 3:** Observed variables' multicollinearity – VIF.

	Consumption	Actual knowledge	Self-perceived knowledge
Cn_1_	2.19		
Cn_2_	–		
Ak_11_		1.05	
Ak_12_		1.07	
Ak_13_		1.16	
Ak_14_		1.04	
Ak_15_		1.24	
Sk_16_			1.03
Sk_17_			–

The values of the latent variables were estimated by means of partial least squares path modelling (PLS) [19] because it is a suitable tool to estimate mixed models with both common factor latent variables, which are caused by their observed variables, and composite ones, which are made up of the related observed variables [17]. Table 4 presents the correlation matrix for the latent variables in which the subsequent latent variables' relationship estimations are based on, in order to analyse consumer behaviour in olive oil markets [20].

**Table 4:** Correlation matrix for the latent variables.

	Consumption (EVOO)	Attitude towards the main product (EVOO)	Attitude towards the product alternative (ROO)	Actual knowledge	Self-perceived knowledge	Brand awareness/associations	Brand perceived quality	Brand loyalty
Consumption (EVOO)	1.00							
Attitude towards the main product (EVOO)	0.55	1.00						
Attitude towards the product alternative (ROO)	−0.41	−0.11	1.00					
Actual knowledge	0.37	0.46	−0.05	1.00				
Self-perceived knowledge	0.19	0.31	0.02	0.38	1.00			
Brand awareness/associations	−0.37	−0.20	0.38	−0.18	−0.04	1.00		
Brand perceived quality	−0.36	−0.24	0.46	−0.20	−0.11	0.60	1.00	
Brand loyalty	−0.30	−0.11	0.36	−0.11	0.03	0.68	0.58	1.00
