# Supplementary material for: Survey data on consumer behaviour in olive oil markets: The role of product knowledge and brand credence
Source: Data Brief. 2018 Apr 30;18:1750–7. doi: 10.1016/j.dib.2018.04.084 (PMC5998222; doi:10.1016/j.dib.2018.04.084)
Supplement: Supplementary file 1 — Supplementary material [file mmc1.docx]

Conflict of interest

All the authors has no conflict of Interset.
